# Uptake of COVID‐19 Vaccines and Intention to Vaccinate in the Democratic Republic of the Congo: A Cross‐Sectional Survey

**DOI:** 10.1002/hsr2.72934

**Published:** 2026-07-31

**Authors:** Marc Bosonkie, Landry Egbende, Nuole Chen, Rawlance Ndejjo, Steven N. Kabwama, Berthold Bondo, Yves Kashiya, Marcel Mubelo, Lily L. Tsai, Mapatano Mala Ali, Rhoda K. Wanyenze

**Affiliations:** ^1^ Department of Nutrition, Kinshasa School of Public Health, School of Medicine University of Kinshasa Kinshasa Democratic Republic of the Congo; ^2^ Department of Political Science Massachusetts Institute of Technology Cambridge Massachusetts USA; ^3^ Department of Disease Control and Environment Health, School of Public Health, College of Health Sciences Makerere University Kampala Uganda; ^4^ Department of Community Health and Behavioural Sciences, School of Public Health, College of Health Sciences Makerere University Kampala Uganda; ^5^ General Hospital of Barumbu Kinshasa Democratic Republic of the Congo; ^6^ Department of Biostatistics and Epidemiology, Kinshasa School of Public Health, School of Medicine University of Kinshasa Kinshasa Democratic Republic of the Congo

**Keywords:** COVID‐19, DRC, intention, uptake, vaccination

## Abstract

**Background and Aims:**

COVID‐19 pandemic caused significant mortality and morbidity locally and globally. Vaccines were introduced as the most effective medical countermeasure against severe morbidity, hospitalization, and death. However, vaccine uptake remains suboptimal in many countries in Sub‐Saharan Africa. This study assessed uptake of COVID‐19 vaccines and intention to vaccinate and associated factors in the Democratic Republic of the Congo.

**Methods:**

We carried out a cross‐sectional survey with data collected from April to May 2022 through mobile phones in the DRC among 1075 persons. Uptake of COVID‐19 vaccines and Intention to vaccinate were the main outcome variables.

**Results:**

Of the participants, 89/1075 (8.3%) declared having received a full dose of the vaccine, and 38/1075 (3.4%) had received an incomplete dose. Results show that the uptake of the COVID‐19 vaccine was associated with being older (adjusted PR = 1.87; 95% CI: 1.05–3.27), living outside of Kinshasa (adjusted PR = 1.77; 95% CI: 1.24–2.53), perceiving the Ministry of Health as competent with regard to the COVID‐19 response (adjusted PR = 1.57; 95% CI: 1.05–2.35), and identifying health workers as a source of information for COVID‐19 (adjusted PR = 1.62; 95% CI: 2.34). Seven out of 10 respondents declared the intention to be vaccinated. Being female (adjusted PR = 0.83; 95% CI: 0.75–0.92), having a high perception in the competence of the MOH staff on COVID‐19 response (adjusted PR = 1.24; 95% CI: 1.13–1.36), and trusting radio as a source of information for COVID‐19 (adjusted PR = 1.11; 95% CI: 1.02–1.22) were factors associated with the intention for COVID‐19 vaccination.

**Conclusion:**

Biological factors (age and sex), perceiving the MOH as competent with regard to the COVID‐19 and sources of COVID‐19 information (radio and health workers) must be taken into account for the development of strategies to improve the uptake of these interventions for COVID‐19 prevention and control.

## 1 Introduction

Infectious diseases have caused enormous consequences worldwide. New pathogenic infections have spiraled into numerous disease outbreaks around the world in recent decades. SARS‐CoV‐2, a new strain of coronavirus originating in Wuhan, China, triggered one of the most severe pandemics worldwide [1]. The healthcare systems in many countries were heavily strained, and the pandemic had a significant economic impact and caused job losses in many industries.

Several interventions were implemented to fight the COVID‐19 pandemic, including non‐pharmacological interventions or NPI [2]. While physical distancing and quarantine slowed the spread of the virus and flattened the epidemic curve, they were not sufficient to completely stop the spread of COVID‐19. Herd immunity acquired through infection or vaccination is a promising option for the population [3].

Different vaccines were developed in 2020, but it took a long time for African countries to access them; and even when they became available, logistical challenges made their distribution difficult [4]. Emphasis was placed on the need to vaccinate people with the WHO‐approved COVID‐19 vaccines. To reduce the burden of COVID‐19 globally and ensure equitable access to COVID‐19 vaccines, the COVAX (COVID‐19 Vaccines Global Access) initiative delivered 1.7 million doses of AstraZeneca's vaccine to the DRC in mid‐March 2021. The vaccination was voluntary and initially targeted healthcare workers and other vulnerable groups, such as people over 55 years of age and those with co‐morbidities (diabetes, high blood pressure, obesity, or any other chronic disease).

The Democratic Republic of the Congo (DRC) was not spared by this pandemic, and the cumulative COVID‐19 cases in DRC were 92,173 with 1390 confirmed deaths (Reuters COVID‐19 Tracker) as of July 2022. The country opted for vaccination to limit the spread of the pandemic, given the challenges to the implementation and adherence to other public health and social measures. Even when vaccine availability improved, only about 55% of the adults were willing to be vaccinated [5]. However, these studies analyzed data from less than half of the country's 26 provinces. These results provided valuable evidence regarding vaccine uptake in DRC, but could not be generalized to the entire country, given the number of provinces included in the sample and the country's great geographical and cultural diversity. This study was conducted to assess factors associated with the uptake of COVID‐19 vaccines and the intention to vaccinate to inform the design of appropriate interventions.

## 2 Materials and Methods

### 2.1 Study Setting

This study was conducted in the DRC, a country in central Africa. The country is subdivided into 26 provinces and comprises 519 health zones. To maintain operational feasibility and provide widespread geographical representation, we segmented the country into six regions based on the mapping of the Belgian Congo (Equateur, Kasai, Katanga, Kivu, Leopoldville, and Orientale) (see Appendix S1).

### 2.2 Study Design and Population

This was a cross‐sectional study conducted from April to May 2022, among a national sample of adults who were selected following the COVID‐19 distribution in the DRC. The study recruited male and female individuals aged 18 years and older from selected regions of the country. Participants eligible for enrollment had to have lived in their current district of residence for at least 6 months. We excluded those who were unable to participate in the interview due to illness or other reasons.

### 2.3 Sample Size Estimation

The sample size for this survey was determined using the formula for cross‐sectional studies [6] with the following assumptions: two‐tailed *Z*‐statistic corresponding to a 95% confidence interval (CI) (1.96) and *p* = 50% as the proportion because the uptake of COVID‐19 vaccines and intention to vaccinate were not known. A significance level *α* equals 5%, and a design effect of 2.5 was considered to account for potential clustering of participants by region and a 10% nonresponse rate. Therefore, we needed 1093 participants for the study.

### 2.4 Sampling Strategy

A sampling strategy similar to the one used in a previously published study [7]. Quotas were set according to age, gender, and location in proportion to the national COVID‐19 case distribution statistics.

- The age distribution was as follows: 18–35 (51%), 36–55 (37%), 56–65 (8%), 65+ (4%).

- Gender: COVID‐19 data from the DRC showed that males accounted for ~63% and females ~37%.

A consultant company was contracted by the Kinshasa School of Public Health to provide a sample of cell phone numbers used within the last 30 days from all 26 provinces of the DRC. The consultant provided the number of unique cell phone numbers needed to conduct 1093 interviews. Based on the preliminary analysis, we anticipated that five cell phone numbers were needed per completed computer‐assisted telephone interview. Thus, a sample of 5465 working cell phone numbers was required. Enumerators then performed systematic calls to verify eligibility criteria: being 18 years or older and having resided in the current region for at least 6 months. Finally, participants were included only if the predefined quotas for their specific age, gender, and regional category remained unfilled.

### 2.5 Data Collection

Before data collection, an independent group of translators validated the translations of the questionnaire (initially in English) using a back translation approach for both French and national languages (Kikongo, Lingala, Swahili, and Tshiluba). The final survey tool in each language was programmed in Survey CTO software. A 3‐day training was organized, where 16 enumerators (4 per national language) and 4 field supervisors (1 per national language) were trained. Trainees had to know French and at least one national language. The training was followed by a 1‐day pre‐test, which identified additional improvements to the questionnaire. A call center was set up at Kinshasa School of Public Health (KSPH) from April 11 to May 6, 2022, where enumerators called survey respondents and recorded responses on electronic tablets between 8 a.m. and 5 p.m. Field supervisors checked the survey data daily to ensure adherence to the established quotas, and conducted quality assurance by back checking 5% of data collected by data collectors. The average interview time was 32 min. Respondents who preferred to defer the phone interviews due to busy schedules or other reasons received follow‐up phone calls based on agreed‐upon appointment times.

### 2.6 Data Management and Analysis

Each interviewer reviewed, edited, and cleaned their data daily during data collection, before uploading it to the server at the end of the day to ensure timeliness and completeness. Data were then downloaded from the server and exported to R 4.0.5 (The R Project for Statistical Computing 4.0.5. GNU GPL v2). Data were collected anonymously, and access to the server was limited to only investigators.

The dependent variables were uptake of COVID‐19 Vaccines and intention to vaccinate. Uptake of COVID‐19 vaccines was assessed by a Yes or No response to the question “Have you received the COVID‐19 vaccine Among those that responded?” Participants were classified as having a full dose if they had received two or more doses of any mRNA vaccine (Pfizer or Moderna) or they had received one dose of the Johnson & Johnson vaccine. They were classified as having an incomplete dose if they had received less than two doses of any mRNA vaccine. In the last case, they were not vaccinated. “No” intention to vaccinate was assessed by a Yes or No response to the question “Do you plan to get vaccinated?” The independent variables were gender, age, religion, education level, wealth index, perceived competence of the MOH, perceived truthfulness of organizations in the COVID‐19 response, trust in academic institutions, trust in government institutions, and sources of information about COVID‐19. Independent variables are described in Appendix S1.

We performed univariate, bivariate, and multivariate analyses. The results were summarized in tables. For the multivariate analyses, we explored the uptake of COVID‐19 vaccines and intention to vaccinate as outcomes, considering a range of independent variables. We used modified Poisson regression models with robust standard errors to estimate adjusted prevalence ratios for the correlations between covariates and the uptake of COVID‐19 vaccines and intent to vaccinate, each specified as a binary outcome. To determine which covariates were kept for the multivariate analysis, we also conducted simple bivariate models, using modified Poisson regression, and selected variables with *p*‐values ≤ 0.20 in the fully saturated model.

### 2.7 Patient and Public Involvement

Neither patients nor other people outside from this study were involved at any steps, including the design, conduct, analyses, and dissemination of these study findings.

## 3 Results

### 3.1 Sociodemographic Characteristics of Participants

In this study, there were 1075 respondents. Overall, 59.8% (643) of the participants were male, and the mean age was 36.4 years (SD ± 12.5). More than half, 61.8% (664) lived in a semi–urban area, while 43.1% (467) had completed secondary school. More than 4 in 10, 41.8% (449) were Pentecostal (Table 1).

**Table 1:** Sociodemographic characteristics of the participants.

Characteristics	Number of participants (*N* = 1075)	Percentage (%)
Sex		
Male	643	59.8
Female	432	40.2
Age (years)		
18–35	554	51.5
36–55	427	39.7
56–64	57	5.3
65+	37	3.4
Mean (SD)	36.4 (± 12.5)	
Region		
Equateur	13	1.2
Kasai	26	2.4
Katanga	137	12.7
Kivu	169	15.7
Leopoldville	677	62.9
Orientale	53	4.9
Residence		
Urban	227	21.1
Rural	184	17.1
Semi‐urban	664	61.8
Education		
Secondary – ordinary	252	23.4
Secondary – advanced	465	43.3
Tertiary or higher	320	29.8
Not stated	38	?%
Religion		
Catholic	261	24.3
Anglican	195	18.1
Born Again/Pentecostal	449	41.8
Muslim	31	2.9
Not stated	139	
Wealth index^a^		
Low SES	338	31.5
Middle SES	403	37.5
High SES	333	31.0
Current occupation		
Unemployed/retiree/housewife	211	19.6
Employed	324	30.1
Self‐employed	256	23.8
Other^b^	101	9.4
Not stated	183	17.0

^a^ The wealth index is calculated as a sum of whether the respondent's household has a television, electricity, computer, sofa, fridge, and cassette/DCD/CD player. Respondents in the high category have 5–6 items, those in the medium category have 3–4 items, and those in the low category have 2 or fewer items.

^b^ Other employment includes: Student, seller, contractor, independent worker, farmer, and so forth.

### 3.2 Vaccination Uptake and Intention to Vaccinate

Of the participants, 8.3% (89) declared having received a full dose of the vaccine, and 3.4% had received an incomplete dose. Among respondents who had not been vaccinated, majority 70.5% (666/948) declared an intention to be vaccinated (Table 2).

**Table 2:** Vaccination uptake and intention to vaccinate among respondents to the survey.

Variable	Count	Percentage (%)
Vaccination uptake		
Full dose	89	8.3
Incomplete dose	38	3.4
Not vaccinated	948	88.2
Vaccination Intention (among unvaccinated)		
Yes	666	70.5
No	282	29.5

### 3.3 Reasons to Vaccinate or Not to Vaccinate

**Figure 1:**
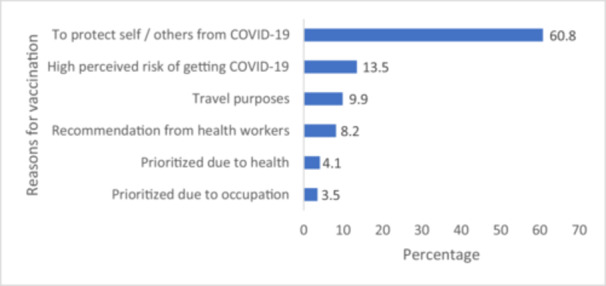
Reasons for vaccination against COVID‐19.

More than half of those who were vaccinated did so to protect themselves from COVID‐19 (60.8%).13.5 perceived themselves to be at high risk of contracting the virus, and 9.9% did so for travel purposes (Figure 1).

### 3.4 Reasons for Not Being Vaccinated Against COVID‐19

**Figure 2:**
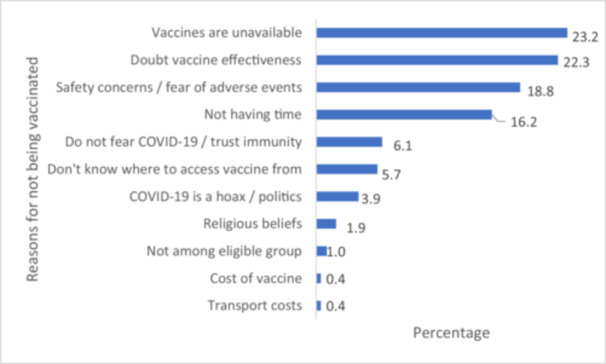
Reasons for not being vaccinated against COVID‐19.

Over 88% (*n* = 948) of the participants had not received a vaccine against COVID‐19. Among the reasons for not receiving the vaccine against COVID‐19, 23.2% said that vaccines were not available, while 22.3% doubted their effectiveness, and 18.8% had safety concerns (Figure 2).

### 3.5 Reasons for no Intention to Vaccinate

**Figure 3:**
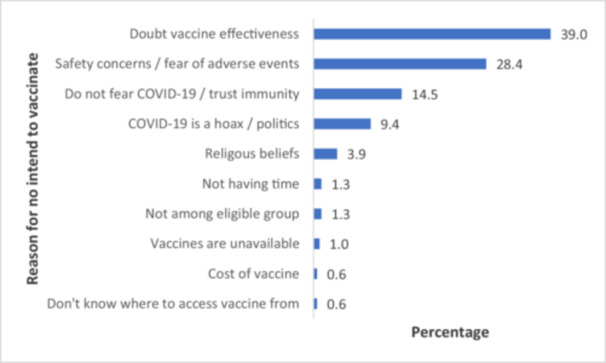
Reasons for no intent to vaccinate against COVID‐19.

Among the reasons for the lack of intention to vaccinate, 39.0% doubted the effectiveness of the vaccine, while 28.4% had concerns with the safety of the vaccine (Figure 3).

### 3.6 COVID‐19 Vaccine Uptake in the DRC

The results of the multivariable analysis show that respondents who were over 56 years of age (adjusted PR = 1.87; 95% CI: 1.07–3.27) and those living outside of Kinshasa (adjusted PR = 1.77; 95% CI: 1.24–2.53) were almost twice as likely to be vaccinated compared to those aged 18 to 35 and those living in Kinshasa, respectively. Respondents with a high perception of the competence of the Ministry of Health (adjusted PR = 1.57; 95% CI: 1.05–2.35) and those who had health workers as a source of information for COVID‐19 also had a high probability of being vaccinated (adjusted PR = 1.62; 95% CI: 1.12–2.34) (Table 3).

**Table 3:** Factors associated with vaccination uptake among Congolese adults.

Variables	Already took COVID‐19 vaccine		Crude PR (95% CI)	*p*	Adjusted PR (95% CI)	*p*
	No	Yes				
Gender						
Male	557 (86.6)	86 (13.4)	1		1	
Female	391 (90.7)	40 (9.3)	0.69 (0.49–0.99)	0.043	0.72 (0.47–1.10)	0.131
Age (years)						
18–35	494 (89.3)	59 (10.3)	1			
36–55	380 (88.9)	47 (11.1)	1.03 (0.72–1.48)	0.866	0.92 (0.61–1.38)	0.685
56+	74 (78.7)	20 (21.3)	1.99 (1.26–3.15)	0.003	1.87 (1.07–3.27)	**0.028**
Region						
Leopoldville	612 (91.2)	59 (8.8)	1			
Other	292 (84.9)	52 (15.1)	1.74 (1.22–2.46)	0.002	1.77 (1.24–2.53)	**0.002**
Education level						
At least secondary – ordinary	410 (88.4)	54 (11.6)	1.26 (0.86–1.86)	0.242	1.33 (0.86–2.07)	0.204
Secondary – advanced	215 (85.3)	37 (14.7)	1			
Tertiary	323 (90.2)	35 (9.8)	0.84 (0.56–1.26)	0.396	0.79 (0.51–1.22)	0.294
Wealth index						
Low SES	290 (85.8)	48 (14.2)	1			
Middle SES	363 (90.1)	40 (9.9)	0.69 (0.47–1.04)	0.075	1.14 (0.75–1.76)	0.538
High SES	294 (88.5)	38 (11.5)	0.81 (0.54–1.20)	0.288	1.42 (0.87–2.31)	0.161
Perceived competence of the MOH						
Low	544 (90.8)	55 (9.2)	1		1	
High	396 (84.8)	71 (15.2)	1.66 (1.19–2.31)	0.003	1.57 (1.05–2.35)	**0.028**
Perceived truthfulness of organizations on COVID‐19 response						
Low	650 (90.3)	70 (9.7)	1		1	
High	277 (83.9)	53 (16.1)	1.65 (1.18–2.31)	0.003	1.34 (0.89–2.01)	0.164
Health workers as information source for COVID‐19						
No	474 (90.9)	47 (9.1)	1		1	
Yes	474 (85.7)	79 (14.3)	1.58 (1.13–2.23)	0.008	1.62 (1.12–2.34)	**0.010**
Local leader as information source for COVID‐19						
No	921 (88.6)	118 (11.4)	1		1	
Yes	27 (77.1)	8 (22.9)	2.01 (1.07–3.79)	0.03	2.01 (0.99–4.04)	0.053
Social media as information source for COVID‐19						
No	706 (87.3)	103 (12.7)	1		1	
Yes	242 (91.3)	23 (8.7)	0.68 (0.44–1.05)	0.081	0.91 (0.58–1.40)	0.659

*Note:* statistical signifiance *p* value less than 0.05.

### 3.7 Factors Associated With Intention to Vaccinate Against COVID‐19

According to the adjusted analysis, female participants had a lower intention to be vaccinated compared to males (adjusted PR = 0.83; 95% CI: 0.75–0.92). Respondents with a high perception of the competence of the MOH on COVID‐19 response (adjusted PR = 1.24; 95% CI: 1.13–1.36) and those with a high perceived truthfulness of organizations on COVID‐19 (adjusted PR = 1.15; 95% CI: 1.05–1.25) had a higher intention to get vaccinated. Respondents with radio as a source of information about COVID‐19 had a higher intention to get vaccinated compared to those who did not have radio as a source of information (adjusted PR = 1.11; 95% CI: 1.02–1.22) (Table 4).

**Table 4:** Intention for COVID‐19 vaccination among Congolese adults.

Variables	Intention for COVID‐19 vaccination		Crude PR (95%CI)	*p*	Adjusted PR (95% CI)	*p*
	No	Yes				
Gender						
Male	130 (23.4)	426 (76.6)	1		1	
Female	151 (38.6)	240 (61.4)	0.80 (0.73–0.88)	< 0.001	0.83 (0.75–0.92)	**< 0.001**
Age (years)						
18–35	161 (32.6)	333 (67.4)	1			
36–55	103 (27.2)	276 (72.8)	1.08 (0.99–1.17)	0.08	1.21 (0.93–1.12)	0.651
56+	17 (22.9)	57 (70.1)	1.14 (0.99–1.31)	0.06	1.05 (0.91–1.21)	0.474
Region						
Leopoldville	190 (30.7)	428 (69.3)	1			
Other	84 (28.8)	207 (71.2)	1.03 (0.94–1.12)	0.56	0.99 (0.91–1.09)	0.980
Education level						
At least secondary – ordinary	68 (31.6)	147 (68.4)	0.95 (0.85–1.06)	0.369	0.96 (0.85–1.07)	0.444
Secondary – advanced	115 (28.1)	294 (71.9)	1			
Tertiary	98 (30.3)	225 (69.7)	0.97 (0.88–1.06)	0.513	1.02 (0.92–1.12)	0.696
Wealth index						
Low SES	67 (23.1)	222 (76.9)	1			
Middle SES	110 (30.3)	253 (69.7)	0.91 (0.83–0.99)	0.04	0.94 (0.87–1.07)	0.548
Hight SES	103 (35.0)	191 (65.0)	0.76 (0.76–0.94)	0.002	0.97 (0.82–1.05)	0.230
Perceived competence of the MOH						
Low	205 (37.7)	338 (62.3)	1		1	
High	71 (17.9)	325 (82.1)	1.32 (1.21–1.43)	< 0.001	1.24 (1.13–1.36)	**< 0.001**
Perceived truthfulness of organizations on COVID‐19 response						
Low	227	423	1		1	
High	45	231	1.29 (1.19–1.39)	< 0.001	1.15 (1.05–1.25)	**0.002**
Radio as information source for COVID‐19						
No	154 (37.2)	259 (62.8)	1		1	
Yes	127 (23.8)	407 (76.2)	1.22 (1.13–1.33)	< 0.001	1.11 (1.02–1.22)	**0.001**
Television as source of information for COVID‐19						
No	83 (25.5)	242 (74.5)	1		1	
Yes	198 (31.8)	424 (68.2)	0.92 (0.84–0.99)	0.038	0.92 (0.83–1.01)	0.088
Trust in academic institutions						
No	191 (32.5)	396 (67.5)	1		1	
Yes	78 (23.8)	250 (76.2)	1.13 (1.04–1.22)	0.004	0.94 (0.86–1.03)	0.173
Trust in government institutions						
No	261 (30)	609 (70)	1		1	
Yes	16 (22.8)	54 (77.2)	1.10 (0.96–1.26)	0.158	1.01 (0.87–1.17)	0.881

*Note:* statistical signifiance *p* value less than 0.05.

## 4 Discussion

This study assessed uptake of COVID‐19 vaccines and intention to vaccinate and associated factors among adults in the DRC. Among respondents, 8.3% reported having received a full dose of the vaccine and 3.4% an incomplete dose. Among the unvaccinated, 7 out of 10 (70.5%) intended to be vaccinated. Among those vaccinated, 8 out of 10 did so to protect themselves against COVID‐19. The results of the multivariable analysis showed that a high perception of the competence of the Ministry of Health, being aged above 56 years, and participants who had received information about COVID‐19 from health workers had a high probability of being vaccinated. Among the reasons for not being vaccinated, 23.2%of respondents said that vaccines were not available, and 21.6% doubted their efficacy. The majority of respondents stated that they intended to be vaccinated during this study. Male participants, those with a high level of trust in organizations fighting COVID‐19 and the Ministry of Health, and those for whom the radio was a source of information about COVID‐19, had a greater intention to be vaccinated than others.

The intention to be vaccinated was higher, while the proportion of people who had been vaccinated remained low. Similar studies have found that the intention to be vaccinated was high during the Delta variant‐related wave [7, 8]. Literature supported that the Delta variant has a high transmissibility rate and increases the risk of hospitalization and death, but is sensitive to vaccines [9]. It should be noted that our study was carried out after the first four COVID‐19 waves, with a cumulative deaths at 1670 [10]. The fear of dying probably played a part in increasing the intention to be vaccinated. In addition, rumors circulating that those not vaccinated would be excluded from their jobs, lose the right to travel, and so forth, could be another explanations of high intention to vaccinate [11].

Our findings showed the unavailability of the vaccine, doubts about its efficacy, and fear of possible vaccine‐related side effects as the main reasons for not vaccinating. These are similar to findings reported in Cameroon [12] and Uganda [7]. Doubts and fear against vaccines are particularly common in low‐ and middle‐income countries, where there is a lower level of trust in foreign pharmaceutical institutions and laboratories [13]. Rumors, such as the belief that Africa's climate prevents the virus from spreading, and concerns about adverse effects [14] contributed to these doubts and to the delay in launching the vaccination campaign in Kinshasa [5]. It will be important to use knowledgeable scientists, community leaders, healthcare providers, and artists of good character to get the right message across to the public, and to do so at regular intervals to avoid the circulation of rumors. Campaigns and risk communication are therefore important to show the benefits of vaccination, especially in a country with low literacy levels, to easily understand the importance of the disease and self‐protection. This understanding of self‐protection turned out to be the main reason why people were vaccinated in our study.

Respondents whose source of information on COVID‐19 was the radio had a higher likelihood of intending to vaccinate. Radio remains one of the most important sources of information in the DRC. Particularly in rural areas [15], most communication takes place via community radios; in the absence of electricity, this is the most important source of information in rural areas [16, 17]. Most of the awareness campaigns carried out, even by health care providers, use this channel to reach a large part of the population. This makes it a source of information that encourages people to seek vaccination. In this way, communication is very important in gaining the population's support for certain health interventions [16, 17].

While respondents who used radio as their source of COVID‐19 information had a higher likelihood of intention to vaccinate, those who obtained information about COVID‐19 from health workers had higher COVID‐19 vaccination rates. Studies have shown that health workers are the most trusted sources for vaccination acceptance [7, 13, 18]. As a key resource in increasing vaccination uptake, health workers should thus be provided with sufficient and accurate information and supported with effective communication tools to encourage their clients at facility and community level to vaccinate [7]. The use of health workers through radio may be a good strategy for increasing uptake. It is demonstrated that when health workers are vaccinated, they are more likely to recommend the same to their clients [19].

Respondents with a high perception of the competence of the MOH on COVID‐19 response had a higher intention to get vaccinated and uptake of the vaccine. It would therefore be important for certain MOH staff, who are seen as competent by the public, to be used to encourage people to get vaccinated.

Findings also showed that women were less likely to intend to vaccinate than men. A study in Uganda found that younger females had a significantly lower vaccination intention when compared to younger males [7]. Rumors were circulating linking getting the COVID‐19 vaccines to infertility in women. It is possible that younger females could have had reproductive health concerns, such as hearing about myths associating vaccines with sub‐fertility, that could have contributed to their vaccine hesitancy [7]. Anti‐myth messages, using health workers, MOH staff considered competent, or communication channels such as radio, are important to improve the intention to vaccinate and the uptake of the vaccine.

## 5 Strengths and Limitations of the Study

The major study's strength resides in its conservative sample size calculation based on a 50% expected proportion. Comparatively, using Uganda's vaccination intention rates available [7, 8] would have required only 350–575 participants, even with a 2.5 design effect. By enrolling 1075 respondents, we ensured high statistical power to estimate the DRC's actual intention and robust subgroup analyses, effectively offsetting the 2.5 design effect. However, there was a coverage bias such that only respondents with mobile phones or those living in areas with network coverage participated in the survey. Furthermore, the timing of our study (April–May 2022) is critical for interpreting the reported vaccination uptake. The 8.3% full vaccination rate observed in our sample is higher than the regional averages reported by the WHO during the same period. This discrepancy may reflect a specific snapshot of the campaign's vaccination progress among the adult population, who were the primary target groups. However, this figure likely represents an overestimation due to the coverage bias; as a phone‐based survey, it reached individuals with better access to communication networks and healthcare information. This selection bias, combined with the concentration of vaccinated individuals in urban areas at that stage of rollout, explains why our figures exceed the national estimates that account for the entire, largely rural, population of the DRC. Similarly, the high intention to vaccinate must be interpreted within the context of the post‐fourth wave period, where perceived risk and social pressure might have packed. Although quotas based on age, sex, and the national distribution of COVID‐19 cases were applied to ensure geographic and demographic representativeness, there was potential for social desirability bias, especially regarding reporting vaccination status. This was minimized by reminding participants that the study was only for research purposes. Also, as a cross‐sectional survey, this study could only determine associations between variables and not causal relationships. On the other hand, our study had a high response rate of the participants (98.3%) consenting to participate, ensuring a more representative survey and higher data quality. Results from the backchecking with the same individuals also showed high consistency with the survey results. The study provides insights into COVID‐19 vaccination uptake and intention to vaccinate, which can facilitate the development of context‐relevant strategies to increase vaccinations.

## 6 Conclusions

COVID‐19 vaccination uptake was low in DRC. Participants in this study generally showed their intention to receive the vaccines. Biological factors (age and sex), region, perceiving the MOH as competent, and sources of COVID‐19 information, such as radio and health workers, must be taken into account for the development of strategies that have the potential to improve the uptake of these interventions for COVID‐19 prevention and control.

## Author Contributions

**Marc Bosonkie:** conceptualization, investigation, methodology, validation, writing – original draft, writing – review and editing, formal analysis, data curation, supervision. **Landry Egbende:** methodology, investigation, validation, writing – review and editing, supervision. **Nuole Chen:** formal analysis, software, project administration, writing – review and editing, methodology, funding acquisition, validation, supervision, data curation. **Rawlance Ndejjo:** writing – review and editing, methodology, funding acquisition, project administration, data curation, supervision. **Steven N. Kabwama:** writing – review and editing, methodology, validation, project administration, supervision. **Berthold Bondo:** supervision, methodology, validation, writing – review and editing. **Yves Kashiya:** methodology, writing – review and editing, validation, supervision. **Marcel Mubelo:** methodology, validation, writing – review and editing, data curation. **Lily L. Tsai:** funding acquisition, writing – review and editing, methodology, validation, project administration. **Mapatano Mala Ali:** funding acquisition, writing – review and editing, methodology, validation, project administration, supervision. **Rhoda K. Wanyenze:** funding acquisition, writing – review and editing, methodology, validation, project administration, supervision.

## Ethics Statement

Ethical approval was obtained from the KSPH ethics committee (Ref: ESP/CE/05/2022). Confidentiality and anonymity were guaranteed. Informed consent was sought from each participant. Before participation in any study activities, respondents were informed of their right to stop the interview at any time if they did not wish to continue, and interviews were conducted in the language they felt most comfortable. All phones and tablets used were password‐protected to protect respondent data, and separate survey‐specific SIM cards were used. Data were also downloaded at the end of each day to the location where the phone calls were made to avoid any risk of losing participants' data.

## Consent

The authors have nothing to report.

## Conflicts of Interest

The authors declare no conflicts of interest.

## Transparency Statement

The corresponding author, Landry Egbende, affirms that this manuscript is an honest, accurate, and transparent account of the study being reported; that no important aspects of the study have been omitted; and that any discrepancies from the study as planned (and, if relevant, registered) have been explained.

## Supporting information


Supporting File

## Data Availability

The data that support the findings of this study are available from the corresponding author upon reasonable request.
