# Supplementary material for: Uptake of COVID‐19 Vaccines and Intention to Vaccinate in the Democratic Republic of the Congo: A Cross‐Sectional Survey
Source: Health Sci Rep. 2026 Jul 31;9(8):e72934. doi: 10.1002/hsr2.72934 (PMC13426016; doi:10.1002/hsr2.72934)
Supplement: Supplementary file 1 — Supporting File [file HSR2-9-e72934-s001.docx]

**Appendix**

Table Corresponding Belgian Congo configuration to current 26 provinces of the DRC

| **Belgian Congo configuration** | **Current provinces configuration** |
| --- | --- |
| Equateur | Equateur  Mongala  Nord Ubangi  Sud Ubangi  Tshuapa |
| Kasai | Kasaï  Kasaï Central  Kasaï Oriental  Lomami  Sankuru |
| Katanga | Haut Katanga  Haut Lomami  Lualaba  Tanganyika |
| Kivu | Maniema  Nord Kivu  Sud Kivu |
| Leopoldville | Kinshasa  Kongo Central  Kwango  Kwilu  Mai Ndombe |
| Orientale | Bas Uele  Haut Uele  Ituri  Tshopo |

OPRATIONNAL DEFINITIONS

### Wealth Index

Variables for the socioeconomic status were consolidated into an additive index. The variables were six including having at the household level television, electricity, computer, sofa set, refrigerator, cassette/CD/DVD player. Government Competence Index: This was assessed by posing the following statement to respondents:

### Truthfulness of Institutions

Truthfulness of Institutions Index was an index based on respondent’s perception of on how truthful the government, Ministry of Health, WHO, scientists, traditional leaders, health workers were with response options of ……….

### Trust in Ministry of Health

Questions for the trust in MOH index include: The Ministry of Health is competent, objective, fair, cosnistent, sincere, faithful, and well-resourced. Response options were Strongly disagree, Partially disagree, Neither agree nor disagree, Partially agree, Strongly agree, Don’t know, Refused. We consolidate these 7 questions into an additive index.

### Trust in Academic Institutions

The 7 questions for the index of trust in academic institutions focus on their competence, objectivity, fairness, consistency, sincerity, loyalty and resources in responding to COVID-19. Response options: Strongly disagree, Partially disagree, Neither agree nor disagree, Partially agree , Strongly agree. These questions are consolidated into an additive index
